# Whole Genome Analyses Suggests that *Burkholderia* sensu lato Contains Two Additional Novel Genera (*Mycetohabitans* gen. nov., and *Trinickia* gen. nov.): Implications for the Evolution of Diazotrophy and Nodulation in the *Burkholderiaceae*

**DOI:** 10.3390/genes9080389

**Published:** 2018-08-01

**Authors:** Paulina Estrada-de los Santos, Marike Palmer, Belén Chávez-Ramírez, Chrizelle Beukes, Emma T. Steenkamp, Leah Briscoe, Noor Khan, Marta Maluk, Marcel Lafos, Ethan Humm, Monique Arrabit, Matthew Crook, Eduardo Gross, Marcelo F. Simon, Fábio Bueno dos Reis Junior, William B. Whitman, Nicole Shapiro, Philip S. Poole, Ann M. Hirsch, Stephanus N. Venter, Euan K. James

**Affiliations:** 1 Instituto Politécnico Nacional, Escuela Nacional de Ciencias Biológicas, 11340 Cd. de Mexico, Mexico; belcha06@yahoo.com.mx; 2 Department of Microbiology and Plant Pathology, Forestry and Agricultural Biotechnology Institute, University of Pretoria, Pretoria 0083, South Africa; marike.duplessis@fabi.up.ac.za (M.P.); chrizelle.beukes@fabi.up.ac.za (C.B.); emma.steenkamp@up.ac.za (E.T.S.); 3 Department of Molecular, Cell, and Developmental Biology and Molecular Biology Institute, University of California, Los Angeles, CA 90095, USA; lbriscoep81@ucla.edu (L.B.); noor.612@gmail.com (N.K.); ehumm@ucla.edu (E.H.); mnarrabit@ucla.edu (M.A.); 4 The James Hutton Institute, Dundee DD2 5DA, UK; Marta.Maluk@hutton.ac.uk (M.M.); marcel.lafos@hutton.ac.uk (M.L.); 5 450G Tracy Hall Science Building, Weber State University, Ogden, 84403 UT, USA; matthewcrook@weber.edu; 6 Center for Electron Microscopy, Department of Agricultural and Environmental Sciences, Santa Cruz State University, 45662-900 Ilheus, BA, Brazil; egross@uesc.br; 7 Embrapa CENARGEN, 70770-917 Brasilia, Distrito Federal, Brazil; marcelo.simon@embrapa.br; 8 Embrapa Cerrados, 73310-970 Planaltina, Distrito Federal, Brazil; fabio.reis@embrapa.br; 9 Department of Microbiology, University of Georgia, Athens, GA 30602, USA; whitman@uga.edu; 10 DOE Joint Genome Institute, Walnut Creek, CA 94598, USA; nrshapiro@lbl.gov; 11 Department of Plant Sciences, University of Oxford, South Parks Road, Oxford OX1 3RB, UK; philip.poole@plants.ox.ac.uk

**Keywords:** *Burkholderia*, *Paraburkholderia*, *Caballeronia*, *Robbsia*, *Mimosa*, *Rhizopus*, symbionts, diazotrophy, root nodulation

## Abstract

*Burkholderia* sensu lato is a large and complex group, containing pathogenic, phytopathogenic, symbiotic and non-symbiotic strains from a very wide range of environmental (soil, water, plants, fungi) and clinical (animal, human) habitats. Its taxonomy has been evaluated several times through the analysis of 16S rRNA sequences, concantenated 4–7 housekeeping gene sequences, and lately by genome sequences. Currently, the division of this group into *Burkholderia*, *Caballeronia, Paraburkholderia*, and *Robbsia* is strongly supported by genome analysis. These new genera broadly correspond to the various habitats/lifestyles of *Burkholderia* s.l., e.g., all the plant beneficial and environmental (PBE) strains are included in *Paraburkholderia* (which also includes all the N_2_-fixing legume symbionts) and *Caballeronia*, while most of the human and animal pathogens are retained in *Burkholderia* sensu stricto. However, none of these genera can accommodate two important groups of species. One of these includes the closely related *Paraburkholderia rhizoxinica* and *Paraburkholderia endofungorum*, which are both symbionts of the fungal phytopathogen *Rhizopus microsporus*. The second group comprises the *Mimosa*-nodulating bacterium *Paraburkholderia symbiotica*, the phytopathogen *Paraburkholderia caryophylli*, and the soil bacteria *Burkholderia dabaoshanensis* and *Paraburkholderia soli*. In order to clarify their positions within *Burkholderia* sensu lato, a phylogenomic approach based on a maximum likelihood analysis of conserved genes from more than 100 *Burkholderia* sensu lato species was carried out. Additionally, the average nucleotide identity (ANI) and amino acid identity (AAI) were calculated. The data strongly supported the existence of two distinct and unique clades, which in fact sustain the description of two novel genera *Mycetohabitans* gen. nov. and *Trinickia* gen. nov. The newly proposed combinations are *Mycetohabitans endofungorum* comb. nov., *Mycetohabitans* *rhizoxinica* comb. nov., *Trinickia caryophylli* comb. nov., *Trinickia* *dabaoshanensis* comb. nov., *Trinickia soli* comb. nov., and *Trinickia* *symbiotica* comb. nov. Given that the division between the genera that comprise *Burkholderia* s.l. in terms of their lifestyles is often complex, differential characteristics of the genomes of these new combinations were investigated. In addition, two important lifestyle-determining traits—diazotrophy and/or symbiotic nodulation, and pathogenesis—were analyzed in depth i.e., the phylogenetic positions of nitrogen fixation and nodulation genes in *Trinickia* via-à-vis other *Burkholderiaceae* were determined, and the possibility of pathogenesis in *Mycetohabitans* and *Trinickia* was tested by performing infection experiments on plants and the nematode *Caenorhabditis elegans*. It is concluded that (1) *T. symbiotica nif* and *nod* genes fit within the wider *Mimosa*-nodulating *Burkholderiaceae* but appear in separate clades and that *T. caryophylli* *nif* genes are basal to the free-living *Burkholderia* s.l. strains, while with regard to pathogenesis (2) none of the *Mycetohabitans* and *Trinickia* strains tested are likely to be pathogenic, except for the known phytopathogen *T. caryophylli*.

## 1. Introduction

*Burkholderia* sensu lato (s.l.) comprise more than 100 species that thrive in several diverse environments [1]. Not long after the initial description of *Burkholderia* by Yabuuchi et al. [2], it was suggested that the genus be divided into several groups [3,4]. Since then, this notion has gathered considerable momentum, with many studies suggesting a formal split between the pathogenic and the plant beneficial environmental (PBE) species on the basis of their core genomes [5]. There has also been opposition to such a split, arguing that the two groups are not distinguished by sufficiently definable and clear phenotypes [6]. Currently, this large genus is divided into *Burkholderia* sensu stricto (s.s.), *Caballeronia, Paraburkholderia*, and *Robbsia andropogonis* [7,8,9].

The division of *Burkholderia* s.l. and the means by which the new genera were initially described has caused skepticism. This was also evident from the minutes of the International committee on systematics of prokaryotes subcommittee for the taxonomy of *Rhizobium* and *Agrobacterium*, which discussed this subject during the 12th Nitrogen fixation Conference held in Budapest, Hungary on 25 August 2016 [10]. The subcommittee stated their position as “Research efforts directed towards robust characterization and taxonomy of *Burkholderia* s.l. species can help in realizing this agricultural potential. Clearly, a large-scale phylogenomic study is required for resolving these taxa”. Therefore, in order to tackle the issue and to settle generic boundaries in *Burkholderia* s.l., a large phylogenomic analysis was carried out using the amino acid and nucleotide sequences of 106 genes from 92 species [11]. The analysis performed with maximum likelihood (ML) unambiguously supported five different lineages: *Burkholderia* s.s., *Caballeronia*, *Paraburkholderia*, *Robbsia andropogonis* and *Paraburkholderia rhizoxinica*.

In this study, an international effort was made to address the generic status of six important species of the *Burkholderia* s.l. assemblage. These are the fungal symbionts, *P. rhizoxinica* and *Paraburkholderia endofungorum* [12], the *Mimosa*-nodulating bacterium *Paraburkholderia symbiotica* [13], the phytopathogen *Paraburkholderia caryophylli* [2], and the soil bacteria *Burkholderia dabaoshanensis* and *Paraburkholderia soli* [14,15]. In 2014, five of these species were transferred to *Paraburkholderia* [7], while the species name, *B. dabaoshanensis*, is still awaiting valid publication. Based on the analysis of the 16S rRNA sequence, all of these taxa formed part of the so-called Transition Group 1 of Estrada-de los Santos in 2016 [4]. The position of these species within the existing phylogenetic framework for *Burkholderia* s.l. was determined using the same phylogenomic approach previously employed by Beukes et al. [11]. Additionally, average nucleotide identity (ANI) [16,17,18] and average amino acid identity (AAI) [19,20,21] values were calculated, together with the analysis of some phenotypic features. Based on these findings, the above-named species belong to two novel genera for which we propose the names *Mycetohabitans* gen. nov. and *Trinickia* gen. nov. (see below).

Although an in-depth analysis of phenotypical differences was out of the scope of this study, in order to address concerns that genome differences alone do not justify the formation of new combinations within *Burkholderia* s.l. [6], the various genomes were consulted in depth for information about differential characteristics. Furthermore, as all of these strains were originally placed in Transition Group 1 between the PBE and the pathogenic *Burkholderia* species [4], two key lifestyles were investigated in more depth: (1) nitrogen fixation, both free-living and symbiotic in association with legumes, which is quite common in *Burkholderia* s.l., particularly in the PBE group [5,22], and (2) the possibility that they may include potential pathogens. In the case of diazotrophy and/or nodulation, we examined the occurrence and phylogeny of essential genes involved in these processes i.e., the nitrogenase enzyme-coding genes, *nifD* and *nifH*, as well as the nodulation genes, *nodABCD*, with particular emphasis on the phylogeny of *Trinickia* vis-à-vis members of the *Burkholderiaceae*. For pathogenesis, the genomes were searched for type III secretion system (T3SS) genes, which encode proteins produced by certain Gram-negative bacteria that are injected into their host, while also performing physiological assays to determine whether or not these strains can infect plants and/or have the ability to kill the nematode *Caenorhabditis elegans*. The presence or absence of sequences for the type IV secretion system (T4SS) used for the transfer of DNA or proteins into a host was also investigated.

## 2. Materials and Methods

### 2.1. Bacterial Strains and Genomes

Of the six species considered in this study, only the genomes for *P. rhizoxinica* HKI 454^T^ and *P. symbiotica* JPY347 were available in the public domain. The genomes for strains in the other five species were determined in this study (Table 1). For this purpose strains of the following species were obtained from various culture collections, *P. symbiotica* (JPY-345^T^, JPY-366 and JPY-581) from the JPY culture collection (housed at the University of York and the James Hutton Institute, Dundee, UK), *B. dabaoshanensis* CCTCC M 209109^T^ (GIMN1.004^T^) from the Agricultural Research Service (ARS) (NRRL B-59553) U.S. Dept. of Agriculture culture collection, and strains of *P. caryophylli* (LMG 2155^T^ = Ballard 720^T^), *P. soli* (GP25-8^T^), and *P. endofungorum* (HKI 454^T^) from the Belgian Coordinated Collection of Microorganisms (BCCM/LMG) culture collection. The type strain of *P. eburnea* (JCM 18070^T^), obtained from the latter collection, was also included, as was *Paraburkholderia rhynchosiae* WSM3937^T^ from the WSM collection at the University of Murdoch, and *Paraburkholderia caribensis* TJ182 from the JPY collection.

The genomes of *P. caryophylli* Ballard 720^T^, *Paraburkholderia eburnea* JCM 18070^T^, *P. endofungorum* HKI 456^T^ and *P. symbiotica* JPY-345^T^ were sequenced by the DOE Joint Genome Institute (JGI) using Illumina technology [23]. An Illumina 300 bp insert standard shotgun library was constructed and sequenced using the Illumina HiSeq–2000 1TB platform (San Diego, CA, USA). All general aspects of library construction and sequencing performed at the JGI can be found at http://www.jgi.doe.gov. All raw Illumina sequence data were filtered using BBDuk [24], which removed known Illumina artifacts and PhiX. Reads with more than one *N* (flanking sequence-dependent N errors) or with quality scores (before trimming) averaging less than 8 or reads shorter than 51 bp (after trimming) were discarded. Remaining reads were mapped to masked versions of human, cat and dog references using BBMAP [24] and discarded if identity exceeded 95%. Sequence masking was performed with BBMask [24]. For assembly, artifact-filtered Illumina reads were assembled using SPAdes (version 3.6.2) [25] and assembly contigs were discarded if the length was < 1 kbp.

The genomes of *P. caribensis* TJ182, *P. caryophylli* LMG 2155^T^, *B. dabaoshanensis* GIMN1.004^T^, *P. rhynchosiae* WSM3739^T^, *P. soli* GP25-8^T^, *P. symbiotica* JPY-366, and *P. symbiotica* JPY-581 were sequenced by MicrobesNG (Birmingham, UK) with the genomic DNA library prepared using the Nextera XT library prep kit (Illumina) following the manufacturer’s protocol with the following modifications: two ng of DNA were used as input and the PCR elongation time was increased to 1 min. DNA quantification and library preparation were carried out on a Microlab STAR automated liquid handling system (Hamilton Robotics, Chicago, IL, USA). Pooled libraries were quantified using the Kapa Biosystems Library Quantification Kit for Illumina on a Roche light cycler 96-qPCR machine (Roche, Geneva, Switzerland). Libraries were sequenced on the Illumina HiSeq using a 250 bp paired-end protocol. Reads were adapter trimmed using Trimmomatic 0.30 with a sliding window quality cutoff of Q15 [26]. De novo genome assembly was carried out with SPAdes (version 3.7) [25] and contigs were annotated using Prokka 1.11 [27]. The genome sizes (contigs) were determined by RAST [28].

### 2.2. Phylogenetic Analysis

The 106-gene amino acid dataset employed for phylogenetic analysis by Beukes et al. [11] was supplemented with the protein sequences for the additional taxa examined in this study (Table S1). Homologous protein sequences were identified and grouped using the Efficient Database framework for comparative Genome Analyses using BLAST score Ratios (EDGAR) server [29]. Individual sequence files were subsequently aligned with MUSCLE [30] as part of CLC Main Workbench 7.6 (CLC Bio, Cambridge, MA, USA). The aligned data sequences were subjected to evolutionary model testing in ProtTest 3.4 [31], followed by concatenation and partitioning in FASconCAT-G v. 1.02 [32]. The partitioned concatenated dataset was subjected to ML analysis with RAxML v. 8.2.1 [33], and branch support was inferred from 1000 bootstrap pseudo-replicates.

### 2.3. Average Nucleotide Identity and Average Amino Acid Identity

As an indication of the relatedness of the taxa investigated, pairwise ANI and AAI values were calculated for the full taxon set on the EDGAR server [29]. For ANI calculations, all shared genomic information was utilized to calculate a similarity value average across homologous regions, bringing into account the sequence similarity as well as the alignment length over homologous regions [34]. AAI calculations were conducted by averaging similarity values for all pairwise homologous protein sequences for each set of two genomes.

### 2.4. Genome-Informed Differential Characteristics

All isolates of *Burkholderia*, *Paraburkholderia* and the proposed genus, *Trinickia*, with available genome sequences included in the study, were compared to identify the potential differences between these genera at the genomic level. Rudimentary analyses were performed by comparing the functional annotations of each of the core genomic components of the respective genera to identify potentially characteristic traits. Core genomes for each genus were calculated with the EDGAR server [29] as described above. This was followed by functional annotation using the Kyoto Encyclopedia of Genes and Genomes (KEGG) [35] following the approach of Palmer and colleagues [36]. Differences between the core genomes were subsequently subjected to the EDGAR server to confirm the presence or absence of genes within the respective members of the three genera.

### 2.5. Analysis of nif and nod Genes

The *nif* and *nod* genes were isolated from the indicated genome sequences with the National Center for Biotechnology Information (NCBI) stand-alone BLAST program by using the corresponding reference genes from *Paraburkholderia phymatum* STM815^T^ (GCF000020045): *nifD* [WP_012406782.1]; *nifH* [WP_012406781.1]; *nodA* [WP_012406745.1]; *nodB* [WP_012406750.1]; *nodC* [WP_012406749.1] and *nodD* [WP_012406751.1]. The genome sequences with a GCF-reference were retrieved from NCBI (https://www.ncbi.nlm.nih.gov/assembly/), and those with an IMG-reference from JGI (https://genome.jgi.doe.gov/).

The homology of the *nif* and *nod* genes to those in the *P. phymatum* STM815^T^ (GCF000020045) reference genome was visualized as a heat map with the R-package ggplot2 v2.2.1 [37] 37 in R v3.4.1 and arranged with Inkscape v0.48. Protein sequences were aligned with MUSCLE v3.8.31 [30,38], and ML phylogenies were inferred with iqTREE v1.5.5 (http://www.iqtree.org) using an iqTREE model-selection [39] and a standard (b 100) nonparametric bootstrap calculation [40]. The phylograms were edited with the R-packages ape v5.0 [41] and ggtree v1.8.2 [42] in R v3.4.1 and arranged with Inkscape v0.48.

### 2.6. Plant Growth Promotion Analysis

The strains were tested for their production of siderophores using Chrome Azurol S casaminoacid (CAS-CAA) medium, while National Botanical Research Institute Phosphate growth (NBRIP) medium was used to test for phosphate solubilization [43]. To determine their ability to fix nitrogen, the strains were grown in a semi-solid Burkholderia-malic acid-glucose-mannitol (BMGM) medium with 20 mg L^−1^ yeast extract added, and then tested for nitrogenase activity using the acetylene reduction assay [44]. The production of indole acetic acid (IAA) was assessed using the method of Jain and Patriquin [45]. The strains were tested on *Mimosa pudica* and siratro (*Macroptilium atropurpureum*) for their ability to nodulate legumes and/or to promote growth according to Elliott et al. [46].

### 2.7. Pathogenicity Tests

For the pathogenicity tests, three different organisms were employed: tobacco (*Nicotiana tabacum* L.), onion (*Allium cepa* L.), and nematodes (*C. elegans*). For the tobacco test, the strains were grown on R2A broth medium for two days at 30 °C with reciprocal shaking (120 rpm). The bacterial culture optical density (OD_600_) was adjusted to 0.5 by dilution with a medium, and 500 μL was injected into the principal vein of a tobacco leaf. The leaf was checked for injury at 48 h. *Pseudomonas savastanoi* pv. *phaseolicola* PsFr-14 and PsFr-96 and *Xanthomonas axonopodis* pv. *phaseoli* XaFr-14 were used as the positive and negative controls, respectively.

In a second plant test, onions were first peeled to remove both the dry external covering and the most external layer without damaging the underlying tissues. The individual onions were quartered with a sterile knife and single onion scales were carefully removed, divided into two lengthwise sections and placed into 90-mm petri plates containing 2 discs of sterile Whatman filter paper no. 1 (Whatman, Los Angeles, CA, USA) using sterile forceps. The filter paper discs covered the entire surface of the petri plates and were pre-moistened with 25 mL of sterile distilled water. Overnight-grown cultures of the different bacterial strains were used in the assay. Individual onion scales were wounded on their inner surface with a sterile pipette tip, and 5 µL of a 10^7^ CFU mL^−1^ culture was inoculated into the wound. The scales were incubated at 30 °C for 72 h. Maceration was rated on a scale described by Jacobs et al. [47]. Each strain was tested three times and an average rating was tabulated. A known onion pathogen strain, *Burkholderia cepacia* 68P128, served as the positive control, while the culture medium alone served as the negative control.

The activity of *C. elegans* fed with different bacterial strains under slow killing conditions was assayed as described by Vílchez et al. [48,49]. Briefly, bacterial strains were spread on two nematode growth media (NGM) plates and incubated at 30 °C for 24 h. Each plate was then seeded with a known number of nematodes from the original control plate (*Escherichia coli* OP50), which was determined using a Zeiss microscope at 10× magnification (Carl Zeiss, Oberkochen, Germany). This number served as a zero-h reading. After counting, the plates were incubated at 24 °C and scored for nematode death every 24 h for 5 days. In all cases, the *E. coli* strain OP50 was a control to estimate the natural death rate of the nematodes, and *Paraburkholderia aeruginosa* PA14 was the positive control for pathogenicity. The experiment was conducted three times with two replicates for each strain.

The evaluation of the effect of bacteria on *C. elegans* was conducted based on the pathogenicity score given by Cardona et al. [50]. The authors established that a given strain could be designated pathogenic for the nematode if one of the following criteria were met: (i) a diseased appearance at day 2, which included reduced locomotive capacity and the presence of a distended intestine; (ii) percentage of live nematodes at day 2 ≤ 50%; and (iii) total number of nematodes at day 5 ≤ 50%. The presence of any one, two, or three of these criteria was scored to differentiate mild from severe infections. A pathogenic score (PS 1, 2, or 3) was given based on the number of criteria met. A strain was considered non-pathogenic when no symptoms of disease were observed (pathogenicity score, PS 0). Additionally, the influence of the bacteria on movement and propagation of the nematodes was monitored for 120 h.

The data are presented as mean ± standard deviation (SD). The statistical analysis was performed using GraphPad Prism software version 5.01 (GraphPad Software, San Diego, CA, USA).

### 2.8. Bioinformatics Analysis of the T3SS

Amino acid sequences for 21 T3SS genes in the *P. rhizoxinica* HKI454^T^ genome were obtained from the DOE-JGI website. This gene set included *sct*, *hpa*, *hrp*, and *araC*-type regulator genes, which were queried using the command line blastp tool from NCBI against a custom database of 10 genomes: *Paraburkholderia caballeronis* LMG 26416^T^, *P. caryophylli* Ballard720^T^, *Paraburkholderia dabaoshanensis* GIMN1.004^T^, *P. endofungorum* HKI456^T^, *P. phymatum* STM815^T^, *P. soli* GP25-8^T^, *P. symbiotica* JPY-345^T^, *P. symbiotica* JPY-347, *P. symbiotica* JPY-581, and *Paraburkholderia tuberum* STM678^T^. With the filtering of blastp hits for sequences with less than 1e-2 e-value and at least 30% identity with *P. rhizoxinica* HKI454^T^, the highest scoring amino acid sequence, if any, from each genome, was used to build a gene tree. Using the MEGA7 (Tokyo Metropolitan University, Tokyo, Japan) ML phylogenetic tree-building algorithm with the Jones–Taylor–Thornton (JTT) model of amino acid substitution, a tree was built for each gene with 1000 bootstrap replications, selecting the tree with the highest log-likelihood [51]. As *hrpB1* and *sctF* were found in fewer than four strains, these genes were excluded from the analysis.

The 21 gene trees with their respective bootstrap values and branch lengths were used to build a single consensus tree using the multi-species coalescent model implemented by ASTRAL-II [52]. For the coalesced tree, the final quartet score was 0.80, representing the percent of quartet trees induced by the 21 input gene trees in the final species trees. The local posterior probabilities displayed on the branches represent the percent of quartets in gene trees that agree with a branch [53].

The analysis for the T4SS was performed using the DOE-JGI, MicrobesNG, and NCBI websites.

## 3. Results and Discussion

### 3.1. Whole-Genome Sequences

The genome features of *P. caribensis* TJ182, *P. eburnea* JCM 18070^T^, *P. rhynchosiae* WSM3937^T^, *Mycetohabitans endofungorum* HKI 456^T^, *Trinickia caryophylli* LMG 2155^T^ and Ballard 720^T^, *Trinickia dabaoshanensis* GIMN1.004^T^, *T. soli* GP25-8^T^, and *Trinickia symbiotica* JPY-345^T^, JPY-366 and JPY-581 are shown in Table 1.

The genome sequences for the type strains of both *M. endofungorum* and *Mycetohabitans rhizoxinica* were markedly smaller than what can be expected for members of *Burkholderia* s.l. Typically, the genome sequences of species in *Burkholderia* s.l. range from 6.0 Mb to 11.0 Mb, with the smallest being 5.8 Mb for *Burkholderia mallei* and the largest being 11.2 Mb for *Paraburkholderia hospita*. In contrast, *M. rhizoxinica* has a genome of 3.8 Mb, while *M. endofungorum* has a genome of 3.3 Mb. This vast difference in genome size can be attributed to the endosymbiotic nature of these species as genome streamlining often occurs in endosymbiotic bacteria [47,54,55].

Overall, the genome sequences for *T. caryophylli*, *T. dabaoshanensis*, *Trinickia soli*, and *T. symbiotica* are comparable in terms of size and G+C content to the remaining members of *Burkholderia* s.l. Amongst the four species, *T. soli* had the smallest genome at 6.1 Mb, while the largest genome was that of *T. dabaoshanensis* at 7.1 Mb. The G+C content of these four species was more similar to that of *Paraburkholderia* and *Caballeronia* (61.9% for *T. soli* to 65.1% for *T. caryophylli*) than that of the higher G+C content reported for *Burkholderia* s.s. [11].

### 3.2. Phylogenetic Analysis

**Figure 1:**
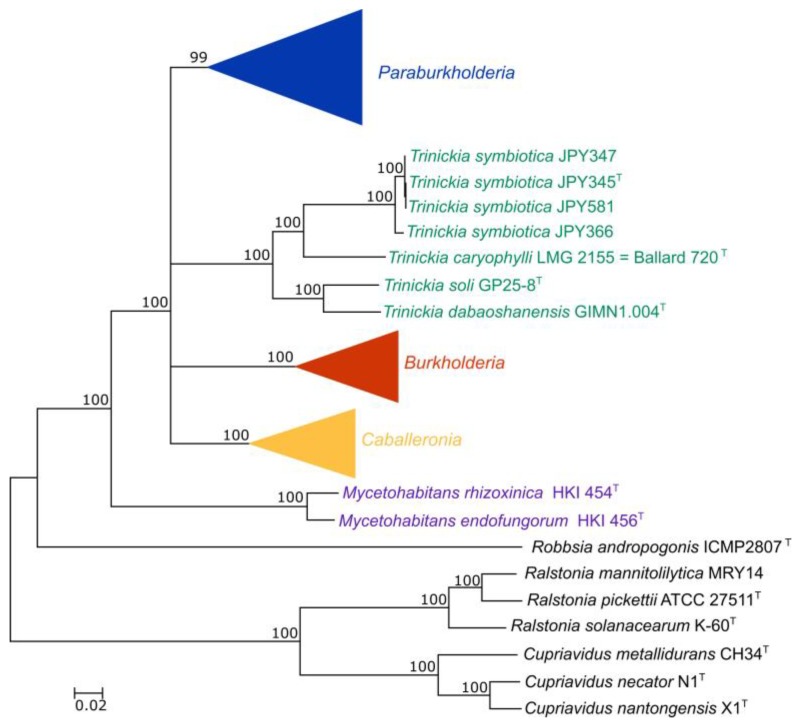
The compressed and collapsed maximum-likelihood (ML) phylogeny of the amino acid sequences of 106 concatenated genes for the 122 strains used in this study of available *Burkholderia* sensu lato genomes showing the positions of the newly-described genera *Mycetohabitans* and *Trinickia* vis-à-vis the previously established genera *Burkholderia*, *Caballeronia*, and *Paraburkholderia*. The scale bar indicates the number of changes per site. All branches with support values below 80% were collapsed to indicate polytomies, as intergeneric relationships depicted by unsupported branching patterns were uncertain.

The concatenated 106-gene dataset of 122 taxa consisted of 27,138 amino acids. ML analysis of the dataset separated the ingroup taxa into five distinct monophyletic groups (Figure 1 and Figure S1). Each of the distinct groups was highly supported with bootstrap values ≥ 95%. These groups corresponded to the genera *Burkholderia* s.s., *Caballeronia* and *Paraburkholderia* as described previously [11], with a further two distinct groups with members currently assigned to *Paraburkholderia*. The first group contained four species (*T. caryophylli*, *T. dabaoshanensis*, *T. soli* and *T. symbiotica*). This group corresponds to *Trinickia* gen. nov., proposed in this study. The second group was sister to all other genera of *Burkholderia* s.l. (except for *Robbsia*) and contained *M. endofungorum* and *M. rhizoxinicia*. This group corresponds to *Mycetohabitans* gen. nov., as proposed in this study. Although the distinctness of these groups from one another was highly supported (reflected by the high branch support values), the relationships between these groups remain unclear, as intergeneric relationships were not supported (collapsed branches had support values of <80%).

### 3.3. Average Nucleotide and Average Amino Acid Identity

**Figure 2:**
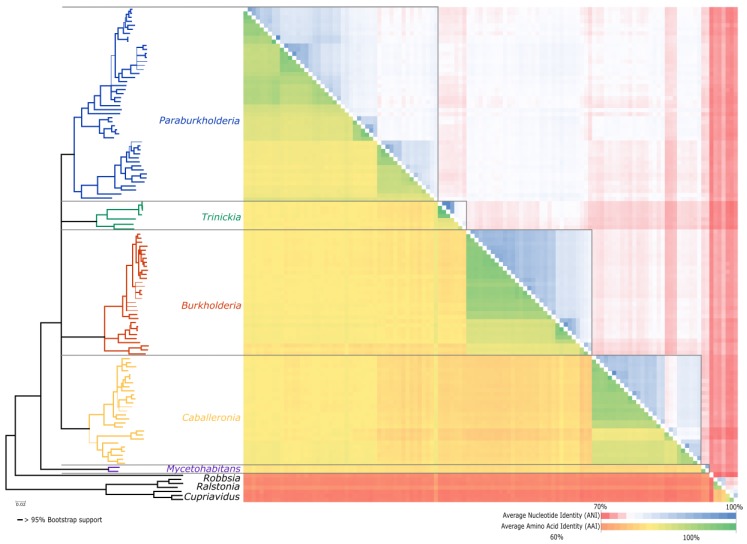
A heat map depicting the average amino acid and nucleotide identity values of the 122 *Burkholderia* sensu lato strains for which whole genomes are available. The cladogram indicating the various intra- and inter-generic relationships were inferred from the amino acid-based ML topology. Average nucleotide identity (ANI) values are indicated in the upper triangle of the map, with average amino acid identity (AVI) values indicated in the lower triangle of the map. For specific values, refer to Table S2.

Based on ANI and AAI calculations (Table S2), the generic groups as recovered in the phylogenetic tree were in overall supported (Figure 2). Intrageneric AAI values were generally comparable, with AAI values for *Paraburkholderia* greater than 74.34%, *Trinickia* greater than 76.74%, *Burkholderia* s.s. greater than 76.88%, and *Caballeronia* greater than 75.8%. For *Mycetohabitans*, only two species are known at this time, and AAI values of 93.35% were obtained for interspecies comparisons. Similarly, intrageneric ANI values for *Paraburkholderia* were greater than 75.18%, the *Trinickia* values were greater than 75.97%, values for *Burkholderia* s.s. were greater than 77.33%, and for *Caballeronia*, values were greater than 75.16%. For *Mycetohabitans*, the interspecies comparison between the two species in this genus resulted in an ANI value of 91.29%.

Based on these analyses, it appears that numerous species are potentially conspecifics, such as *P. sediminicola* and *P. terricola*, although some high values for well-differentiated species such as *B. mallei* and *B. pseudomallei* were also obtained. There was, however, a clear separation between the monophyletic groups where individuals within a genus were generally more closely related to each other than to individuals outside of each genus. Both *M. endofungorum* and *M. rhizoxinica* are assigned to the novel genus *Mycetohabitans* gen. nov. since they share a 91% ANI but less than 80% ANI to members of any other genus.

### 3.4. Genome-Informed Differential Characteristics

As a means to investigate the distinctness of the proposed genus *Trinickia* from *Burkholderia* and *Paraburkholderia*, genome comparisons were conducted to identify potential biologically informative differences between the gene content of members of these genera (Table S3). These genomic comparisons consisted of basic functional comparisons between the respective core genomes to identify metabolically important differences. Based on these genomic comparisons, some genes were found to be present in the majority of members of a genus as opposed to absent in the members of another genus or vice versa. Examples of these differences were genes for benzoate degradation (present in all members of *Paraburkholderia*), starch and sucrose metabolism (present in all members of *Paraburkholderia* and *Trinickia*), glycerolipid metabolism (present in all members of *Trinickia*), cysteine and methionine metabolism (absent in all members of *Trinickia* and present in all members of *Burkholderia*), and d-arginine and d-ornithine metabolism (present in all members of *Burkholderia*). Based on these initial analyses, it appeared that all members of *Paraburkholderia* possess the ability to metabolize 4-hydroxybenzoate, whereas *Paraburkholderia* and *Trinickia* can metabolize starch to amylose, while *Burkholderia* have the ability to utilize additional amino acids. The main differential phenotypic features of the type species all the genera in the family Burkholderiaceae, including *Mycetohabitans* and *Trinickia*, are given in Table 2.

### 3.5. nif and nod Gene Analysis

**Figure 3:**
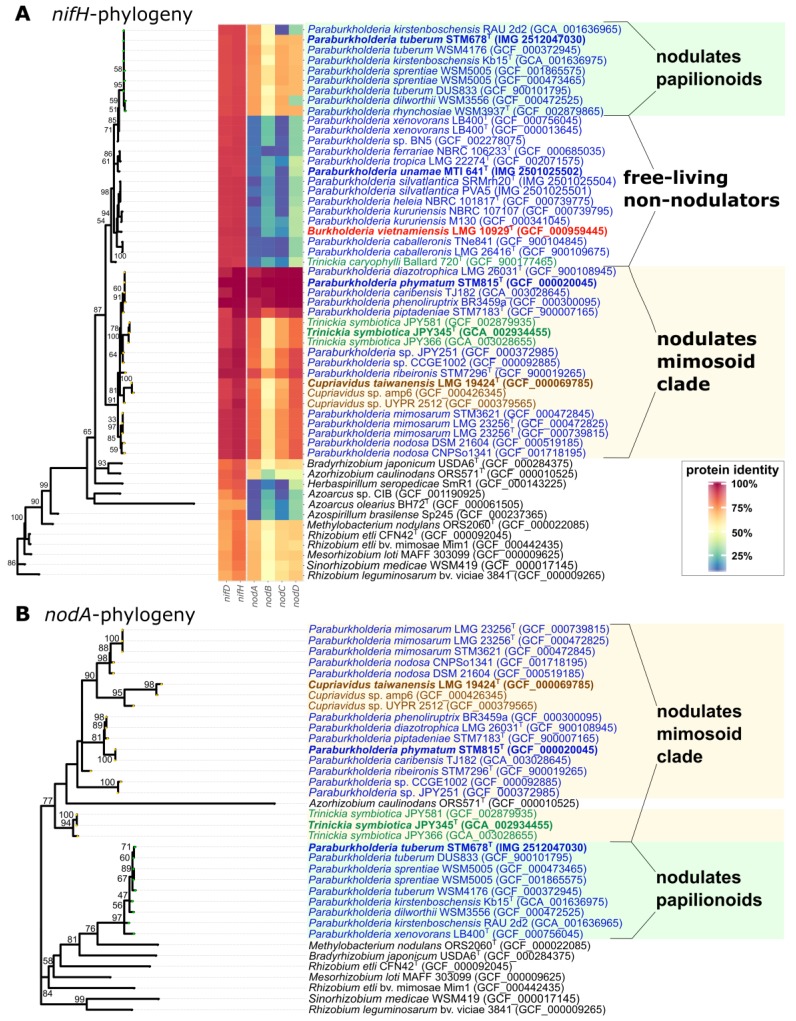
The ML phylogenies of *Burkholderiaceae* species using sequences of *nifH* (**A**) and *nodA* genes (**B**) inferred with iqTREE and using 100 nonparametric bootstrap calculations. Only bootstrap values greater than 50 are shown. α-Proteobacteria are labeled in black, *Paraburkholderia* in blue, *Burkholderia* in magenta, *Cupriavidus* in brown and *Trinickia* in green. In the *nifH* phylogram, non-nodulating species of bacteria harboring nitrogen-fixing genes, but no nodulation genes, are labeled as free-living non-nodulators (this group is absent in the *nod*-gene phylogeny). In both phylograms, the group of bacteria specifically nodulating papilionoid legumes is indicated with green shading, and the group specifically nodulating mimosoids is indicated with yellow shading; note that both the *nifH* and *nodA* gene phylogenies reveal similar grouping of nodulating strains in accordance with their indicated host specificity. Colors in the heatmap correspond to the percent identity of protein sequences to the *nif* and *nod* genes of *Paraburkholderia phymatum* STM815^T^, which was used as the reference genome; color gradient from blue (0%) to green (25%), yellow (50%), orange (75%) and red (100%).

Phylogenies were constructed from full-length sequences of the *nifH* (Figure 3A), *nifD* (Figure S2A), *nodA* (Figure 3B), *nodB* (Figure S2B), *nodC* and *nodD* (Figure S3A,B) genes obtained from the genomes of the strains featured in the present study. First, it should be noted that *nif* was not detected in any of the *Caballeronia*, *Mycetohabitans* or *Robbsia* species. It was widely present in *Paraburkholderia* and in free-living/plant-associated species, such as *P. tropica* and *P. xenovorans*, and in legume symbionts (e.g., *P. phymatum*). It was also present in *Burkholderia contaminans*, *B. lata* and *B. vietnamiensis*, [56,57], but it was absent in most *Burkholderia* s.s. species. In terms of phylogeny, *nif* genes were highly conserved across the *Burkholderiaceae*, including *Cupriavidus*, and both genes analyzed had a similar topology (Figure 3A and Figure S2A). This showed that the *nif*-containing *Burkholderiaceae* strains in the genera *Burkholderia*, *Cupriavidus*, *Paraburkholderia* and *Trinickia* constituted a large and separate cluster from other diazotrophs in the β-proteobacteria, such as *Azoarcus* and *Herbaspirillum*, and were separate from plant-associated and symbiotic diazotrophs in the α-proteobacteria, such as *Azospirillum* and *Rhizobium* s.l. This further suggests that *nif* in the *Burkholderiaceae* has a different evolutionary origin from other symbionts/plant-associated bacteria, including those in the β-proteobacteria, which we might have assumed had a similar origin. However, this may simply reflect the likelihood that *nif* in the β-proteobacteria has been acquired from a number of sources via horizontal gene transfer; e.g., this has clearly happened with *Azoarcus*, both branches of which (represented in Figure 3A and Figure S2A by *A. olearius* BH72 and *Azoarcus* sp. CIB) have *nif* from separate origins [58].

Within the *Burkholderiaceae*, it was previously noted from a smaller number of complete genome sequences [59] that the *nif* genes were divided into three clades: (1) free-living diazotrophs (*Burkholderia*, *Paraburkholderia*), (2) *Paraburkholderia* strains, which nodulate diverse papilionoid legumes native to the Fynbos biome of the South African Cape region (this group is derived from the free-living diazotrophs) and (3) strains of *Paraburkholderia* and *Cupriavidus*, which nodulate legumes within the mimosoid clade native to the Americas (*Mimosa* and its close relatives in the *Piptadenia* Group). The present study using several more genomes confirms this division, but also shows that the *T. symbiotica* strains are clearly in a sub-clade of the mimosoid-nodulating clade. However, the *nif* genes of *T. caryophylli* are more closely related to those of the free-living clade and occupy a basal position within this group (Figure 3A).

The *nod* gene phylogenies also exhibit a similar organization to that described by de Meyer et al. [59], i.e., the papilionoid-nodulating *Paraburkholderia* strains are in a separate clade to the mimosoid-nodulating *Paraburkholderia* and *Cupriavidus* strains. Although both β-proteobacterial clades are distinct from nodulating α-proteobacteria, the *nod* genes of the papilionoid-nodulating strains appear to be derived from the α-rhizobia, particularly *Methylobacterium nodulans* and *Bradyrhizobium* (Figure 3B, Figures S2B and S3A,B). However, this is clearly not the case with the mimosoid-nodulating *Paraburkholderia* strains and related genera, whose *nod* genes are highly divergent from α-rhizobia, suggesting a very different evolutionary origin. The *nod* genes of the newly-sequenced strains *P. diazotrophica*, *P. piptadeniae* and *P. ribeironis*, isolated from *Mimosa* and *Piptadenia* species in South America [60,61,62], are all in the mimosoid-nodulating clade, as expected, as is *P. caribensis* TJ182 (isolated from invasive *Mimosa* in Taiwan), which is actually identical to *P. phymatum* in both *nif* and *nod* genes (Figure 3A,B, Figures S2A,B and S3A,B). However, within the mimosoid-nodulators, the *T. symbiotica* strains isolated from *Mimosa* species in Brazil [13] occupy a separate lineage that is comparable to that occupied by *Cupriavidus* (Figure 3B). This suggests that *T. symbiotica* is not so closely related to other *Mimosa*-nodulating β-rhizobia in terms of its *nod* genes, and this may be connected to their host, *M. cordistipula*, which is a rare endemic species whose habitat is confined to the highland (>900 m) *campos rupestres* environments of the Chapada Diamantina in northeast Brazil [13,63]. The only other reported host of *T. symbiotica* is *M. misera* [13], which is a widespread species in North East Brazil and occurs at a wider range of altitudes than *M. cordistipula*. It remains to be seen if rhizobial strains isolated from *M. misera* are similar to JPY345^T^ and JPY-581, in terms of their *nod* genes. With further regard to other potential hosts of *T. symbiotica*, a recent study of symbionts from another genus in the mimosoid clade, *Calliandra*, which is native to the same sites as *M. cordistipula* and *M. misera*, was only nodulated by *Paraburkholderia*. This suggests that *Calliandra* may not nodulate with *Trinickia* [64].

The *nif* and *nod* gene comparisons within the *Burkholderiaceae* are summarized in the heatmap in Figure 3 which uses *P. phymatum* STM815^T^ as a reference genome. It is hypothesized that geographical separation of the two nodulating β-rhizobial clades (American vs. African) have led to the separate evolution of their *nod* genes. Therefore, they have very different host ranges and do not have an ability to nodulate each other’s hosts [5,65]. However, this is not strictly true. An interesting feature of β-rhizobial strains in the mimosoid clade, such as *P. phymatum* and *P. nodosa*, is that they also often nodulate promiscuous papilionoid legumes in the tribe *Phaseoleae*, including common bean (*Phaseolus vulgaris*), cowpea (*Vigna unguiculata*), siratro (*Macroptilium atropurpureum*) and *Dipogon lignosus* [66,67,68]. With specific regard to *Trinickia*, Lardi et al. [69] have recently shown that *T. symbiotica* JPY-345^T^ was unable to nodulate any of these promiscuous legumes. This reinforces the idea that *nod* genes in *T. symbiotica* are functionally as well as genetically different from other mimosoid-nodulating β-rhizobia, and hence have a greatly restricted host range, being confined to *Mimosa* species [69,70]. Host range studies like those undertaken with *P. phymatum* STM815^T^ [71] and *P. tuberum* STM678^T^ [65] are needed to establish if this is indeed the case.

### 3.6. Nodulation and Plant Growth Promotion Features

In nodulation tests with *Mimosa pudica*, plants inoculated with the *T. symbiotica* strains JPY-345^T^, JPY-366, and JPY-581 formed nodules, but not those with JPY-347. No nodules were formed on plants infected by *M. endofungorum*, *M. rhizoxinica*, *T. caryophylli*, *T. dabaoshanensis* or *T. soli*. In the case of *T. caryophylli*, it rapidly killed the inoculated *Mimosa* plants, but the other non-nodulating strains either had no effect on *Mimosa* growth or slightly enhanced it, suggesting that they might be plant growth-promoting rhizobacteria (PGPR). *Mycetohabitans endofungorum* HKI 456^T^, *M. rhizoxinica* HKI 454^T^, *T. caryophylli* LMG 2155^T^, *T. dabaoshanensis* GIMN1.004^T^, *T. soli* GP25-8^T^, *T. symbiotica* JPY-345^T^ and *T. symbiotica* JPY-581, were accordingly tested for PGPR activities. All strains produced siderophores, except for *M. rhizoxinica*. Only *T. dabaoshanensis* was able to solubilize phosphates. Synthesis of IAA was carried out by each strain, but the level of production was lower than the control strain *Azospirillum brasilense* SP7^T^. *Trinickia caryophylli* was able to fix nitrogen under free-living conditions, which was previously shown [4], but the other strains were unable to (Table S4). This is in contrast to the ability of *T. symbiotica* to fix nitrogen symbiotically in nodules (Reference [13] and this study).

### 3.7. Virulence Tests

*Mycetohabitans rhizoxinica* HKI 454^T^, *M. endofungorum* HKI 456^T^, *T. symbiotica* JPY-345^T^ and JPY-581, *T. caryophylli* LMG 2155^T^, *T. dabaoshanensis* GIMN1.004^T^, and *T. soli* GP25-8^T^ were tested for their effects on tobacco leaves. *T. caryophylli* caused water-soaked lesions and a loss of leaf tissue integrity (Figure S4), and *T. soli* and *T. dabaoshanensis* elicited small water-soaked lesions, a response not recorded previously for these bacteria. The other tested strains, except for the positive controls in H-I, caused no ill effects on tobacco leaves (Figure S4).

In trial experiments using entire pearl onion bulbs, *B. gladioli* BSR3 resulted in reduced biomass accumulation and increased tissue browning and maceration compared to *P. tuberum* STM678^T^ (not shown). To determine whether or not the *Trinickia* strains exhibited any pathogenic potential, we used a bona fide onion pathogen, *B. cepacia* 68P128, on a detached onion bulb scale assay [45]. Table 3 and Figure S5 show that the *B. cepacia* strain induced the greatest amount of tissue damage (score of 3) whereas *B. caryophylli* Ballard720^T^ was less virulent (score of 2). In contrast, none of the other tested strains were pathogenic, including *M. rhizoxinica* HKI 454^T^.

*Caenorhabditis elegans* tests are frequently used to analyze broad-host range microbial pathogenicity, with *Pseudomonas aeruginosa* PA14 included as a positive control as it is an effective killer of nematodes. On NGM, *C. elegans* exposed to PA14 were motile, but avoided the bacteria, which remained unconsumed by the nematodes leading to their death (Table S5) in contrast to the normal food source *E. coli* OP50. None of the tested strains exhibited an inhibitory effect on *C. elegans* motility, except for *M. rhizoxinica* HKI 454^T^, *T. caryophylli* Ballard 720^T^, and *T. dabaoshanensis* GIMN1.004^T^, where decreased motility was observed after 24–48 h (Table S5). Additionally, feeding with *M. rhizoxinica* HKI 454^T^ and *T. caryophylli* Ballard 720^T^ resulted in significantly lower numbers of adult worms. The worm populations in these treatments were similar to that of the *P. aeruginosa* PA14 (positive control) treatment (Figure S6). When grown on *M. rhizoxinica*, the nematodes did not digest the bacteria and eventually starved (Table S4). *Mycetohabitans* species synthesize rhizonin, a cyclopeptide important for plant diseases caused by its fungal host *Rhizopus microsporus* [72], but the mechanism by which it kills *C. elegans* is not known. In NGM medium, in which *P. caballeronis* LMG 26416^T^ and the *Trinickia* species (with the exception of *T. caryophylli* Ballard 720^T^) grew, *C. elegans* nematodes were motile and digested the bacterial lawn by 72 h, although the feeding behavior was altered. In the case of *T. caryophylli* Ballard 720^T^, worm motility slowed after 24 h. In comparison to the *C. elegans* population fed with *E. coli* OP50, the presence of *T. symbiotica* JPY581, *T. symbiotica* JPY347, *T. dabaoshanensis* GIMN1.004^T^, and *P. caballeronis* LMG 26416^T^ resulted in a reduction of 40%, 28%, 31% and 36% of the nematode population, respectively (Figure S6).

Earlier it was noted that 12 different environmental and symbiotic *Burkholderia* species completely lacked the virulence-associated T3SS-3, which is essential in pathogenic species for infecting mammals [73]. Although the T3SS influences host range in the rhizobium-legume symbiosis, to our knowledge, no evidence exists so far that this secretion system affects host range in the nodulating β-rhizobia.

**Figure 4:**
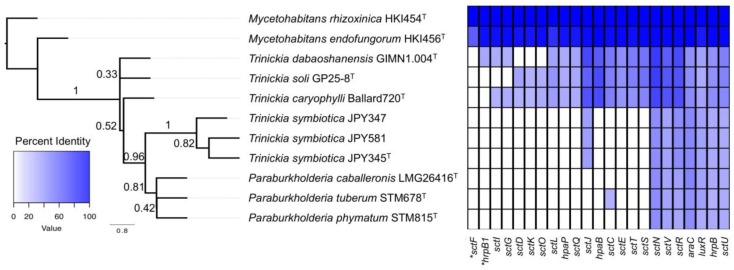
The type III secretion system genes in *Mycetohabitans, Paraburkholderia*, and *Trinickia* strains. The columns of the heatmap correspond to the percentage identity of protein sequences of type III secretion system genes in *M. rhizoxinica* HKI454^T^. At left is the unrooted consensus tree indicated by 21 gene trees with a final quartet score of 0.799. Displayed at the branch points are the support values for the quadripartition as determined by Astral-II. *The gene *sctF*, found in only 2 genomes, was not used to build the tree; *hrpB1*, only found in 3 genomes, was also excluded.

We analyzed the T3SS in several *Burkholderia* species and strains using *M. rhizoxinica* T3SS genes to query the other species. *Paraburkholderia* species are very different from the two pathogenic *Mycetohabitans* species, and the T3SS genes are not well conserved between the species (Figure 4). In *P. caballeronis* LMG 26416^T^, *P. phymatum* STM815^T^, and *P. tuberum* STM678^T^, the *sctNVURTS* genes [74] are, in most cases, more similar to flagellar biosynthesis proteins. SctN most significantly aligned to a flagellum-specific ATPase, whereas SctV [74] aligned with the flagellar biosynthesis protein FlhA. SctR aligned to the flagellar biosynthesis protein FliP, and SctU to the flagellar biosynthesis protein FlhB.

The *T. symbiotica* strains lacked most of the T3SS genes, and those that were present lacked similarity to the genes of *Mycetohabitans* species (Figure 4). *Trinickia caryophylli, T. dabaoshanensis*, and *T. soli* possessed a more complete T3SS, but were missing the *sctF* and *hrpB1* genes, which are also not found in rhizobial species [74]. For these species, additional T3SSs may exist based on the presence of multiple gene copies. As described above, a gene encoding SctU is homologous to genes in *M. rhizoxinica* (46%) and *Xanthomonas campestris* (57%), with a second gene in *X. campestris* (55%) and has homologs in *Yersinia enterocolitica* (35%) and *Escherichia albertii* (30%). In contrast, *Mycetohabitans* species appear to have all the components of a functional T3SS, including *sctF*, which encodes the needle monomer, and *hrpB1*/*hrpK*, which is required for Hrp pilus formation. Both are found in phytopathogens such as *X. campestris pv. vesicatoria* strains (Figure 4). Although *T. dabaoshanensis* and *T. soli* did not kill *C. elegans* or affect onion leaves, *T. caryophylli* killed *C. elegans*, onion tissue, and *Mimosa pudica*, and elicited water-soaked lesions on tobacco leaves. It is unclear which mechanism(s) *T. caryophylli* employs for pathogenesis in these organisms, but it is likely to be independent of the T3SS. Interestingly, *T. soli* and *T. dabaoshanensis* induced the formation of water-soaked lesions on tobacco leaves. It is possible that other pathogenic strategies or alternate secretion systems are involved in this response.

In our earlier analysis of the T4SS [73], genes encoding an intact gene cluster in the *Burkholderia* strains studied were not detected. Similarly, a gene cluster that is homologous to the T4SS cluster of *Agrobacterium tumefaciens* C58 was not detected in *M. endofungorum* HKI456^T^, *M. rhizoxinica* HKI454^T^, *T. caryophylli* Ballard720^T^, or *T. soli* GP25-8^T^. Moreover, our result for *M. rhizoxinica* HKI454^T^ contrasts with an earlier report, which indicated that a T4SS was present [75]. Although *M. rhizoxinica* has a gene annotated as a limited host range *virA* protein, the orthologous genes in *T. caryophylli* Ballard720^T^ and *M. endofungorum* HKI456^T^ are annotated simply as a “signal transduction histidine kinase”. Moreover, the gene neighborhood bears no resemblance to that of the *A. tumefaciens* T4SS gene cluster.

The genomes of *T. dabaoshanensis* GIMN1.004 and the *Mimosa* nodule strains *T. symbiotica* JPY-345^T^, *T. symbiotica* JPY-347, *T. symbiotica* JPY-366, and *T. symbiotica* JPY-581 have clusters of genes orthologous to *A. tumefaciens vir* genes, as well as to genes involved in conjugal transfers, such as *tra* and *trb*. However, the *vir* genes are not necessarily in the same operon as is the case for *A. tumefaciens*. Some, but not all of the *T. symbiotica* species and *T. dabaoshanensis* have sequences related to *virB1, virB2*, as well as *virB3*, *virB4*, *virB5*, *virB6*, *virB8*, *virB9*, *virB10, virB11*, and *virD4*. *T. symbiotica* JPY345^T^, *T. symbiotica* JPY347, *T. symbiotica* JPY581, and *T. dabaoshanensis* GIMN1.004 have the most complete operons. Nevertheless, many of the genes annotated as *vir* showed very low % identity to *Agrobacterium* genes. More importantly, absent from all the gene clusters in this group are genes orthologous to the sensor histidine kinase *virA* or the response regulator *virG*. Although it is possible that these *vir*-like operons are regulated by a different two-component system, the absence of *virA*/*G* and other *vir* genes, as well as genes more closely related to conjugal transfer genes, strongly suggests that the *Trinickia* genomes do not have a T4SS involved in virulence.

### 3.8. Description of New Genera

#### 3.8.1. Description of *Mycetohabitans* gen. nov.

○ *Mycetohabitans* (My.ce.to.ha’bi.tans. Gr. n. *mykês*, *etos*, fungus; L. pres. part. *habitans* inhabiting; N.L. fem. n. *Mycetohabitans* inhabitant of fungi).

Characteristics for this genus were derived from the literature [12]. Cells are Gram-negative, short, motile rods. Oxidase and catalase positive. Colonies are very small, flat, circular, and cream colored. The growth on media is very poor. It grows in an aerobic or microaerophilic atmosphere, but not under anaerobic conditions. Growth is observed between 16–45 °C. β-Galactosidase negative. Positive for the utilization of glycerol, but glucose is not metabolized.

The type species of the genus is *Mycetohabitans rhizoxinica*.

- Description of *Mycetohabitans rhizoxinica*

*Mycetohabitans rhizoxinica* (rhi.zo.xi’ni.ca. N.L. n. *rhizoxinum* rhizoxin; L. f. suff. -*ica* suffix used with various meanings; N.L. fem. adj. *rhizoxinica* referring to the ability of this organism to produce the antimitotic agent rhizonin).

Basonym: *Paraburkholderia rhizoxinica* [7].

The description for the species is provided in Partida-Martinez et al. [12]. Based on phylogenetic analysis of 106 conserved protein sequences, high support is obtained for the placement of this species into the novel genus *Mycetohabitans*.

The type strain of the species is HKI 454^T^ (= DSM 19002^T^ = CIP 109453^T^).

- Description of *Mycetohabitans endofungorum*

*Mycetohabitans endofungorum* (en.do.fun.go’rum. N.L. pref. *endo*- from Gr. *endon* within; L. gen. pl. n. *fungorum* of fungi; N.L. gen. n. *endofungorum* referring to the endosymbiotic nature of this organism with fungi).

Basonym: *Paraburkholderia endofungorum* [7].

The species description is provided in Partida-Martinez et al. [12]. The phylogenetic placement of this species into the novel genus *Mycetohabitans* is highly supported based on the concatenation of 106 conserved protein sequences.

The type strain of the species is HKI 456^T^ (=DSM 19003^T^ = CIP 109454^T^).

#### 3.8.2. Description of *Trinickia* gen. nov.

○ *Trinickia* (Tri.nick’i.a. N.L. fem. n. *Trinickia* formed after M.J. Trinick, an Australian microbiologist who was the first to isolate β-rhizobia from *Mimosa*).

All characteristics for this genus were derived from the literature [2,13,14,15]. Cells are Gram-negative, aerobic, non-spore-forming rods. Growth occurs between 10–40 °C for all members of this genus. Most members are catalase positive with the exception of *T. dabaoshanensis*. Positive for the hydrolysis of Tween 40 and 80. Positive for the utilization of *N*-acetyl-d-glucosamine, l-arabinose, d-fructose, L-fucose, α-d-glucose, d-mannitol, d-sorbitol, pyruvic acid methyl ester, succinic acid, bromosuccinic acid, l-alanine, l-alanylglycine, and l-asparagine. Compounds utilized by most members within the genus are d-arabitol, adonitol, d-galactose, *myo*-inositol, d-mannose, d-raffinose, l-rhamnose, succinic acid mono-methyl-ester, cis-aconitic acid, citric acid and formic acid.

The type species for the genus is *Trinickia symbiotica*.

- Description of *Trinickia symbiotica* comb. nov.

*Trinickia symbiotica* (sym.bio’ti.ca. N.L. fem. adj. *symbiotica* from Gr. n. *symbios*, a companion, partner, living together, symbiotic).

Basonym: *Paraburkholderia symbiotica* [7].

The species description is provided in Sheu et al. [13]. Phylogenetic analysis based on 106 conserved protein sequences provided high support for the placement of this species in the novel genus *Trinickia*.

The type strain of this species is JPY345^T^ (=LMG 26032^T^ = BCRC 80258^T^).

- Description of *Trinickia caryophylli* comb. nov.

*Trinickia caryophylli* (ca.ry.o.phyl’li. N.L. masc. n. *caryophyllus*, specific epithet of *Dianthus caryophyllus*, carnation; N.L. gen. n. *caryophylli*, of the carnation.).

Basonym: *Paraburkholderia caryophylli* [7].

The description of this species is provided in Yabuuchi et al. [2]. Phylogenetic analysis based on the concatenation of 106 conserved protein sequences indicates a high support for the inclusion of this species in the new genus *Trinickia*.

The type strain of this species is LMG 2155^T^ (=ATCC 25418^T^ = CFBP 2429^T^ = JCM 10488^T^).

- Description of *Trinickia dabaoshanensis* comb. nov.

*Trinickia dabaoshanensis* (da.bao.shan.en’sis. N.L. *dabaoshanensis*, fem. adj. pertaining to Dabaoshan, South China, where the type strain was isolated).

Basonym: *Burkholderia dabaoshanensis* [14].

The description of the species is provided in Zhu et al. [14]. Concatenated phylogenetic analysis of 106 conserved protein sequences places this species into the novel genus *Trinickia* with high support.

The type strain of the species is GIMN-1.004^T^ (= CCTCC M 209109^T^ = NRRL B-59553^T^ = LMG 30479^T^).

- Description of *Trinickia soli* comb. nov.

*Trinickia soli* (so’li. L. gen. n. *soli*, of soil, the source of the type strain).

Basonym: *Paraburkholderia soli* [7].

The description of this species is provided in Yoo et al. [15]. Phylogenetic analysis of 106 conserved protein sequences indicates the placement of this species into the new genus *Trinickia* with high support.

The type strain of the species is GP25-8^T^ (=KACC 11589^T^ = DSM 18235^T^).

## 4. Conclusions

The present study revealed the existence of two new genera within *Burkholderia* s.l.: *Mycetohabitans* as a genus containing fungal symbionts with small genomes, and *Trinickia* as a diverse genus containing plant-associated and soil bacteria. The analyses of genes and activities involved in N_2_-fixation, legume symbiosis, and pathogenicity did not reveal any particular patterns in the new combinations vis-à-vis *Burkholderia* s.l., except that *T. symbiotica* was clearly divergent from other nodulating (para)burkholderias in terms of its *nif* and *nod* genes, and *T. caryophylli* has *nif* genes, which are basal to free-living diazotrophic burkholderias. Moreover, uniquely among the *Trinickia* species, *T. caryophylli* is a phytopathogen. Neither of the other *Trinickia* species (*B. dabaoshanensis* and *B. soli*) is diazotrophic, symbiotic or pathogenic, and this perhaps reflects the “mosaic” nature of such lifestyles, which in itself is such a common and intriguing feature across the *Burkholderiaceae* [22].

We believe that *Mycetohabitans* and *Trinickia* are robust genera based upon the genome comparisons and on the analysis of genes specific for metabolism and/or certain lifestyles, but we also accept that *Burkholderia* s.l. continues to experience taxonomical changes due to the analysis of more sequenced genomes, and the present study may undergo revision as more becomes known about *Burkholderia* s.l. taxonomy. Indeed, the frequent release of genomic information provides unparalleled breakthroughs for evaluating taxonomic relationships among microorganisms. A cursory examination of ANI and AAI values suggests that based on the lower similarity values typically associated with existing genera, *Burkholderia* s.s., *Paraburkholderia*, and *Trinickia* gen. nov. in particular, could theoretically be further divided into several other new genera. However, we would caution against proceeding too rapidly (if at all) in this direction without first establishing that any new combinations also have some functional biology differentiating them.

## Figures and Tables

**Table 1:** The genome sequencing statistics for *Burkholderia* sensu lato strains sequenced in this study.

Statistic	*Paraburkholderia eburnea*	*Paraburkholderia rhynchosiae*	*Mycetohabitans endofungorum*	*Trinickia caryophylli*	*Trinickia caryophylli*	*Trinickia dabaoshanensis*	*Trinickia soli*	*Trinickia symbiotica*	*Trinickia symbiotica*	*Trinickia symbiotica*	*Paraburkholderia caribensis*
**Strain**	JCM 18070^T^	WSM3937^T^	HKI 456^T^	LMG 2155^T^ = Ballard 720^T^	Ballard 720^T^ = LMG 2155^T^	GIMN1.004^T^	GP25-8^T^	JPY 345^T^	JPY 581	JPY 366	TJ182
**Sequencing Centre**	DOE Joint Genome Institute	MicrobesNG	DOE Joint Genome Institute	DOE Joint Genome Institute	MicrobesNG	MicrobesNG	MicrobesNG	DOE Joint Genome Institute	MicrobesNG	MicrobesNG	MicrobesNG
**Sequencing Platform**	Illumina HiSeq-2000 1TB	Illumina HiSeq 2500	Illumina HiSeq-2000 1TB	Illumina HiSeq-2000 1TB	Illumina HiSeq 2500	Illumina HiSeq 2500	Illumina HiSeq 2500	Illumina HiSeq-2000 1TB	Illumina HiSeq 2500	Illumina HiSeq 2500	Illumina HiSeq 2500
**NCBI taxonomy ID**	1,189,126	487,049	417,203	28,094	28,094	564,714	380,675	863,227	863,227	863,227	75,105
**NCBI BioProject ID**	PRJNA369942	PRJNA427925	PRJNA370785	PRJNA369920	PRJNA427926	PRJNA427927	PRJNA427928	PRJNA369937	PRJNA427929	PRJNA445642	PRJNA445638
**Number of reads**	6,886,312	1,204,873	7,561,076	7,357,578	962,962	828,393	918,663	6,294,534	2,076,457	1,180,541	809,533
**Assembly method**	SPAdes	SPAdes	SPAdes	SPAdes	SPAdes	SPAdes	SPAdes	SPAdes	SPAdes	SPAdes	SPAdes
**Sequencing coverage**	149.1X	58.8X	348.7X	169.1X	56.4X	47.6X	61.6X	149.1X	131.3X	66.2X	35.9X
**N50**	294,829	226,289	213,816	480,986	187,187	186,667	231,363	252,951	255,942	387,494	89,490
**L50**	7	12	6	6	13	13	10	9	9	7	31
**Largest contig [bp]**	983,800	527,307	365,500	792,225	401,224	433,345	514,473	819,300	663,178	786,277	294,652
**Number of contigs**	58	181	76	49	161	104	105	61	121	57	242
**Genome size [bp]**	6,947,977	8,032,361	3,288,408	6,543,652	6,581,896	7,093,755	6,096,514	6,714,023	6,753,015	7,005,740	9,206,228
**G+C content**	64.09%	61.74%	61.27%	64.72%	64.72%	63.28%	62.98%	63.00%	63.01%	63.00%	62.49%
**Assembly Accession Number**	GCA_002917095.1	GCA_002879865.1	GCA_002927045.1	GCA_900177465.1	GCA_002879875.1	GCA_002879885.1	GCA_002879855.1	GCA_002934455.1	GCA_002879935.1	GCA_003028655.1	GCA_003028645.1

**Table 2:** The differential phenotypic features among the type species of all the genera in the family *Burkholderiaceae*.

	Feature	Plant Pathogen	N-Fixation	Chitinolytic Activity *	Predator Bacterium	Cell Type	Fungus Endosymbiont	Legume Nodulation	Obligately Endosymbiont	NO_3_ to NO_2_	Growth at >60 °C	OL-1	OL-2
Species													
*Burkholderia cepacia* J2315^T^		+	− **	−	−	Rods	−	−	−	−	−	+	+
*Caballeronia glathei* ATCC 29195^T^		−	+	−	−	Rods	−	−	−	nf	−	nd	nd
*Chitinimonas taiwanensis* cf^T^		nd	−	+	−	Rods	−	−	−	+	−	nd	nd
*Cupriavidus necator* N-1^T^		−	− **	−	+	Short rods	−	− **	−	+	−	−	−
*Lautropia mirabilis* AB2188^T^		nd	−	−	−	Coccoid	−	−	−	+	−	nd	nd
*Limnobacter thiooxidans* CS-K2^T^		nd	−	−	−	Rods	−	−	−	−	−	nd	nd
*Mycetohabitans rhizoxinica* HKI 454^T^		+	−	−	−	Coccoid rods	+	−	−	nd	−	nd	nd
*Pandoraea apista* LMG 16407^T^		nd	−	−	−	Rods	−	−	−	−	−	nd	nd
*Paraburkholderia graminis* C4D1M^T^		−	−	−	−	Rods	−	−	−	+	−	nd	nd
*Paucimonas lemoignei* A62^T^		nd	+	−	−	Rods	−	−	−	−	−	nd	nd
*Polynucleobacter necessarius* ATCC 30859^T^		nd	−	−	−	Rods	−	−	+	nd	−	nd	nd
*Ralstonia pickettii* ATCC 27511^T^		+	−	−	−	Rods	−	−	−	+	−	−	−
*Robbsia andropogonis* LMG 2129^T^		+	−	−	−	Rods	−	−	−	−	−	−	−
*Thermothrix thiopara* ATCC 29244^T^		nd	nd	−	nd	Rods	nd	nd	−	+	+	nd	nd
*Trinickia symbiotica* JPY345^T^		−	+	−	−	Rods	−	+	−	+	−	−	−

* Use of chitin as the exclusive carbon, nitrogen, and energy source for growth, both under aerobic and anaerobic conditions. **, The type strain does not have the activity but other strains have the feature. nd: data not determined. nf: data not found. OL: Ornithine lipid. The feature information was taken from the original description. ATCC, American Type Culture Collection.

**Table 3:** The pathogenicity of the *Mycetohabitans* and *Trinickia* strains on onion bulb scales (*Allium cepa* L.) compared with a bacterial strain known to be pathogenic (*Burkholderia cepacia* 68P128).

Strains	Rating for the Degree of Tissue Maceration after 72 h
Control	0
*B. cepacia* 68P128	3 (67–100% macerated tissue area)
*M. rhizoxinica* HKI 454^T^	0
*T. symbiotica* JPY 581	0
*T. symbiotica* JPY 366	0
*T. symbiotica* JPY 347	0
*T. caryophylli* Ballard 720^T^	2 (34–66% macerated tissue area)
*T. soli* GP25-8^T^	0
*T. dabaoshanensis* GIMN1004^T^	0
*P. caballeronis* LMG 26416^T^	0

Onions treated with culture medium alone served as negative controls. Individual onion scales were wounded on their inner surface with a sterile pipette tip, and 5 µL of a 10^7^ CFU mL^−1^ culture was inoculated into the wound. The scales were incubated at 30 °C for 72 h. Maceration was rated on a scale described by Jacobs et al. [45]. Data are means ± SD of three replicates.

## Supplementary Materials

The following are available online at http://www.mdpi.com/2073-4425/9/8/389/s1, **Table S1**: Sources and accession numbers for all the *Burkholderia* (s.l.) genomes used in this study. **Table S2**: Average nucleotide identities (ANI) and average amino acid identities (AAI) calculated using all the *Burkholderia* (s.l.) and *Cupriavidus* genomes available for this study. **Table S3**: Genome-informed differential characteristics of *Burkholderia* s.s., *Paraburkholderia Mycetohabitans* and *Trinickia* strains with particular focus on genes involved in metabolism. **Table S4**: Activities of plant growth promotion (PGP) traits of *Mycetohabitans* and *Trinickia* strains. **Table S5**: Pathogenicity score and behavioral response of *Caenorhabditis elegans* to *Mycetohabitans* and *Trinickia* strains. **Figure S1**: A maximum-likelihood phylogeny of the amino acid sequences of 106 concatenated genes for the 122 strains used in this study of available *Burkholderia* (s.l.) genomes. **Figure S2**: Maximum-likelihood phylogenies of *Burkholderiaceae* species using sequences of *nifD* (A) and *nodB* genes (B) inferred with iqTREE and using 100 nonparametric bootstrap calculations. **Figure S3**: Maximum-likelihood phylogenies of *Burkholderiaceae* species using sequences of *nodC* (A) and *nodD* genes (B) inferred with iqTREE and using 100 nonparametric bootstrap calculations. **Figure S4**: Hypersensitivity test on tobacco leaves. **Figure S5**: Pathogenicity of *Mycetohabitans* and *Trinickia* strains on onion bulb scales (*Allium cepa* L.) compared with bacteria known to be pathogenic (*Burkholderia cepacia* 68P128). **Figure S6**: Survival and growth of *Caenorhabditis elegans* on culture plates of *Mycetohabitans* and *Trinickia* strains compared with bacteria known to be harmless (*E. coli* OP50) and pathogenic (*Pseudomonas aeruginosa* PA14) to the worms.
